# Plasticity in zwitterionic drugs: the bending properties of Pregabalin and Gabapentin and their hydrates

**DOI:** 10.1107/S2052252519004755

**Published:** 2019-05-22

**Authors:** U. B. Rao Khandavilli, Matteo Lusi, Patrick J. Frawley

**Affiliations:** a Synthesis and Solid State Pharmaceutical Centre (SSPC), University of Limerick, Limerick, Ireland; b Department of Chemical Science and Bernal Institute, University of Limerick, Limerick, Ireland; c Department of Mechanical, Aeronautical and Biomedical Engineering, University of Limerick, Limerick, Ireland

**Keywords:** plastic bending, hydrates, single-crystal transformations

## Abstract

The investigation of mechanical properties in molecular crystals is emerging as a novel area of interest in crystal engineering. Indeed, good mechanical properties are required to manufacture pharmaceutical and technologically relevant substances into usable products. In such endeavour, bendable single crystals help to correlate microscopic structure to macroscopic properties for potential design. The hydrate forms of two anticonvulsant zwitterionic drugs, Pregabalin and Gabapentin, are two examples of crystalline materials that show macroscopic plasticity. The direct comparison of these structures with those of their anhydrous counterparts, which are brittle, suggests that the presence of water is critical for plasticity. In contrast, structural features such as molecular packing and anisotropic distribution of strong and weak interactions seem less important.

## Abstract 2

The hydrate forms of the zwitterionic drugs Pregabalin and Gabapentin show plastic bending in a single crystal, whereas the anhydrous equivalents are brittle. A close structural analysis attempts to justify such behaviour.

## 1. Introduction   

In recent times, the classic idea of crystals as a brittle objects has been progressively abandoned (Dunitz, 1984 ▸). Observation of phenomena such as shearing (Reddy *et al.*, 2005*a* ▸) or bending (Reddy *et al.*, 2005*b* ▸) as well as martensitic (Zamir *et al.*, 1994 ▸; Steiner *et al.*, 1993 ▸; Ding *et al.*, 1991 ▸; Etter & Siedle, 1983 ▸), super-elastic (Takamizawa & Miyamoto, 2014 ▸) and super-plastic (Takamizawa *et al.*, 2018 ▸) transformations clearly show that organic crystals share many similarities with metallic and covalent inorganic analogues. Mechanical properties of molecular crystals are investigated and discussed with increasing frequency and those studies have attracted considerable attention (Reddy *et al.*, 2010 ▸; Naumov *et al.*, 2015 ▸). Aside from the understandable academic curiosity, mechanical properties are important for the manufacturing of crystalline solids into products. For example, in the pharmaceutical industry, good plasticity guarantees that microcrystalline drug substances can be readily manufactured into tablets without the addition of excipients (Thoorens *et al.*, 2015 ▸; Chang & Sun, 2017 ▸).

A characteristic of molecular crystals is that they are held together by supramolecular interactions. It follows that crystal synthesis occurs in relatively mild conditions, whilst a supramolecular approach enables a certain degree of structure design (Desiraju, 1989 ▸; Etter, 1990 ▸; Moulton & Zaworotko, 2001 ▸). In this view, understanding how molecular features determine a crystal structure and, in turn, material properties would enable crystal engineering and properties design.

The rapid scientific advances of the past decades allow the confident prediction of how a given set of molecules will pack in a crystal structure (Reilly *et al.*, 2016 ▸). On the other hand, a general theory is still missing to infer bulk chemical and mechanical properties from structures. Therefore, material development proceeds through the application of a series of practical rules, which often apply to specific types of materials (Lusi, 2018 ▸; Corpinot & Bučar, 2019 ▸).

The first attempts to rationalize the relationship between structure and mechanical properties in molecular crystals involved small aromatic molecules (Reddy *et al.*, 2006*b* ▸,*a* ▸). In such materials, aromatic stacking and halogen or hydrogen interactions coexist in the same structure and extend along different crystallographic axes. Manipulation of face-indexed single crystals showed that bending occurred only perpendicular to the planes of the weaker interactions, whereas the stronger supramolecular bonds helped to preserve structural integrity in the other directions (Saha *et al.*, 2018 ▸). Then, chemical anisotropy appeared as a requirement for plasticity and such features were thought to guarantee the realization of bendable single- and multi-component crystals (Krishna *et al.*, 2016 ▸; Rao Khandavilli *et al.*, 2017 ▸; Saha & Desiraju, 2017 ▸; Alimi *et al.*, 2018 ▸; Nath *et al.*, 2018 ▸). However, the discovery of plastic bending in the (quasi)-isotropic structures of di­methyl sulfone proves that alternative mechanisms might exist (Thomas *et al.*, 2017 ▸).

**Figure 1:**
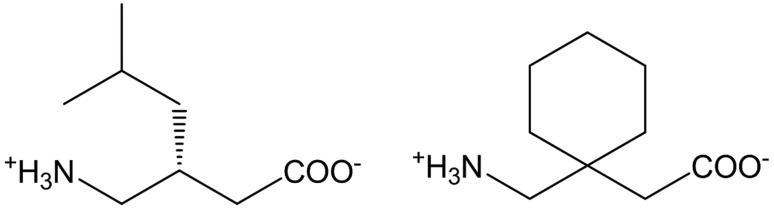
Molecular structure of Pregabalin (left) and Gabapentin (right).

Due to their relevance in manufacturing and processing, many studies of mechanical properties involve APIs (active pharmaceutical ingredients), which suggest that hydrate forms might have higher plasticity than their corresponding anhydrates (Sun & Grant, 2004 ▸; Liu *et al.*, 2018 ▸; Fucke *et al.*, 2012 ▸; Chang & Sun, 2017 ▸). In these cases, the mechanical properties were only observed at the microscopic level on polycrystalline powders, making the correlation between structure and properties elusive. In *p*-hy­droxy­benzoic acid (Sun & Grant, 2004 ▸) and uric acid (Liu *et al.*, 2018 ▸), despite creating a rigid 3D hydrogen-bonded network, the inclusion of water enables the separation of zigzag chains that are interdigitated in the anhydrous forms (see Figs. S10 and S11 of the supporting information). Hence, increased plasticity was explained by the removal of a mechanical (or steric) obstacle to molecular movement, which was deemed necessary for plastic bending. Anhydrous theophylline shows a different story: hydrogen bonds, π stacking and weak C=O⋯H bonds support the structure along the orthogonal crystallographic directions, and there is no mechanical interlock to prevent molecular movement (Fucke *et al.*, 2012 ▸; Chang & Sun, 2017 ▸). In that view, crystals of theophylline should be highly plastic. Instead, plasticity is lower than in the hydrate form, which is characterized by 2D hydrogen-bonded networks that pack in an interdigitated zigzag fashion (see Fig. S12 of the supporting information). In the theophylline system, increased plasticity was attributed to the ‘lubricant’ action of water molecules that allows easier slippage of the 2D sheets. Ultimately, also in the case of hydrate forms, multiple mechanisms explain plastic bending and the observation of such a phenomenon on single crystals could give new insights into the role of water. This work reports on the mechanical properties in the anhydrous and hydrate forms of Pregabalin and Gabapentin (Fig. 1 ▸). Despite the similar crystal packing, the hydrate forms show superior plasticity, which results in macroscopic bending of single crystals whereas the anhydrous forms are brittle.

## 2. Experimental   

Pregabalin (SPG) and Gabapentin (GP) were purchased from Flourochem. Methanol was purchased from Sigma–Aldrich and Milli-Q water was used for the experiments.

*Crystallization of SPG*: SPG crystals were obtained from commercially available SPG dissolved in methanol to saturation and left at room temperature for 2 days.

*Crystallization of SPGH I*: SPG was dissolved in pure water and crystallized at 280 K for 3–5 days to obtain good-quality crystals.

*Crystallization of GP and GPH:* GP and its hydrate crystals are reproduced by following the same procedure mentioned in the literature (Wang *et al.*, 2017 ▸; Reece & Levendis, 2008 ▸).

*Optical microscopy:* single crystals of SPGH I and GPH were selected and bent with tweezers and a needle, the images were captured using an Olympus IX53 microscope under 4× magnification.

*IR spectroscopy:* IR spectra for SPG and SPGH I were collected on a PerkinElmer Spectrum 100 F T-IR Spectrometer equipped with a PerkinElmer Universal ATR Sampling Accessory.

*Raman spectroscopy:* Raman spectra for SPG and SPGH I were collected on a Horiba Jobin Yvon LabRam Aramis spectrometer with a 532 nm laser source. The spectrometer was coupled with an Olympus BX40 confocal microscope with a CCD camera cooled by a thermoelectric Peltier device. Raman maps were processed using the *LabSPEC 5* software package.

*Powder X-ray diffraction:* Powder X-ray diffraction data were collected on an Empyrean diffractometer (PANalytical, Philips) using Cu *K*α_1,2_ radiation (λ = 0.1541 nm) at room temperature operated at 40 kV and 40 mA. The samples were scanned over the range 4–40° 2θ using a step size of 0.02° 2θ and a scan speed of 0.02° 2θ s^−1^.

*Differential scanning calorimetry:* calorimetric measurements of SPG and SPGH I were performed on a DSC 214 Polyma, NETZSCH instrument. Typically, 3–5 mg of sample was accurately weighed into a hermetically sealed aluminium pan and heated to 250 °C at a 10 °C min^−1^ heating rate under a nitro­gen gas flow of 40 ml min^−1^.

*Thermogravimetric analysis:* Thermograms of SPG and SPGH I were measured with a Perkin-Elmer TGA 4000 instrument at a heating rate of 10 °C min^−1^ under a nitro­gen stream of 20 ml min^−1^.

*X-ray crystallography:* single-crystal data for SPGH I were collected at ambient temperature using a three-circle Bruker D8 Quest diffractometer with a sealed tube Mo anode, *K*α radiation = 0.71073 Å and a Photon 100 detector. Single-crystal data for SPGH II were collected at 150 K under a nitro­gen-flow (Oxford Cryosystem) using a three-circle Bruker D8 Quest with microfocus Cu anode, *K*α radiation = 1.5418 Å and a Photon 100 detector.

The data were integrated and corrected for absorption with the Bruker Apex Suite. The structure solution was obtained by direct methods and refined against all *F* ^2^ with the *SHELX* software interfaced though *X-SEED* (Barbour, 2001 ▸). Non-hydrogen atoms were refined anisotropically and hydrogen atoms were placed in calculated positions, refined using idealized geometries (riding model) and assigned fixed isotropic displacement parameters.

## 3. Results and discussion   

**Figure 2:**
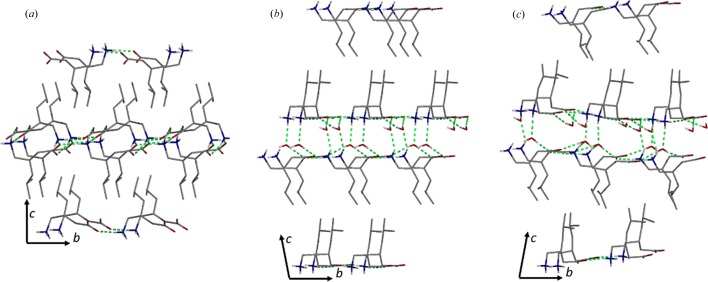
Schematic representation of the crystal packing of (*a*) SPG, (*b*) SPGH form I and (*c*) SPGH form II. Hydrogen bonding interactions are in green, selected hydrogen atoms have been omitted for clarity.

Amino ­acids are an important class of biologically active molecules that exist as zwitterionic tautomers under neutral conditions. Among them, (*S*)-3-iso­butyl-γ-amino­butyric acid, or Pregabalin (SPG), is an anticonvulsant blockbuster drug. In the anhydrous *P*2_1_2_1_2_1_ crystal, which has the refcode CIDDEZ (Venu *et al.*, 2007 ▸) in the CSD (Groom *et al.*, 2016 ▸), the zwitterion dipoles pack along the *b* axis generating a double layer that is coplanar to the (110) plane. The structure is supported by a 2D network of charge-assisted hydrogen bonds between the ammonium and carboxyl­ate groups [Fig. 2 ▸(*a*)]. As multiple layers stack on top of each other, the iso­butyl groups interdigitate along the [001] direction. The crystals are brittle, as expected for mechanically interlocked structures.

Recrystallization of SPG in water at 280 K results in the monohydrate form SPGH I. Single-crystal analysis reveals a monoclinic *C*2 polar structure (Table 1 ▸). The zwitterions are arranged along the [010] axis and are intercalated by water molecules so that the double molecular layers separate into parallel planes. At the same time, the molecules in each layer come closer together preventing interdigitation [Fig. 2 ▸(*b*)].

SPGH I is unstable at room temperature, reverting to the anhydrous SPG phase in air (Fig. S6). As expected from the different crystal packing, the dehydration is associated with a loss of macroscopic crystallinity. SPGH I undergoes a polymorphic transition upon cooling below 150 K. The phase transition is reversible (enantiotropic) and occurs in a single-crystal-to-single-crystal fashion, as demonstrated by variable-temperature single-crystal diffraction experiments. The packing of the low-temperature SPGH II phase is virtually identical to the high-temperature phase. The differences are limited to a reduced symmetry (*Z*′ = 16) and a different arrangement of the hydrogen-bonded bridges with water [Fig. 2 ▸(*c*)].

In molecular materials, the single-crystal-to-single-crystal reduction of symmetry on cooling is known. Often, such transformations assume the aspect of an order–disorder transition, whereby a certain thermal energy is required for a molecule to rotate or vibrate between symmetry-related crystallographic positions (Lusi & Barbour, 2013 ▸; Braga *et al.*, 2018 ▸). Here, the change occurs between two fully ordered structures instead. In that view, the preserved crystallinity is evidence of the high plasticity of these phases, and suggests that the supramolecular bonds with water can be easily broken and reformed without affecting the rest of the structure.

**Figure 3:**
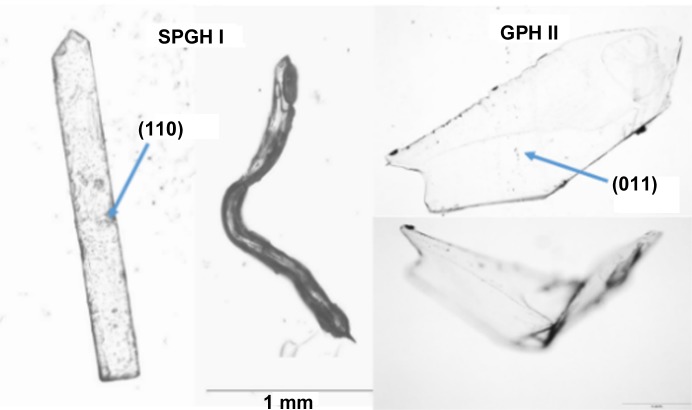
Single crystals of SPGH I and GPH II before and after the mechanical deformation. The larger crystal faces are indexed.

Indeed, crystals of SPGH I exhibit plastic bending on the application of a moment perpendicular to the *c* axis (Fig. 3 ▸). Hence, the bending mechanism involves the sliding of successive layers along the crystallographic *b* direction (Fig. 2 ▸). In order for this to happens, either the hydrogen bond between water and zwitterions or the dispersion forces between the non-polar-layer intermolecular bonds need to quickly break and reform; crystallographic studies cannot determine which are responsible for the bending.

GP is a molecular and pharmaceutical analogue of SPG. In GP, cyclo­pentane substitutes the iso­butyl group but the supramolecular feature of the two molecules are essentially equivalent. Due to extensive polymorph screening, multiple anhydrous and hydrate forms of GP are reported (Braga *et al.*, 2008 ▸; Reece & Levendis, 2008 ▸; Wang *et al.*, 2017 ▸).

**Figure 4:**
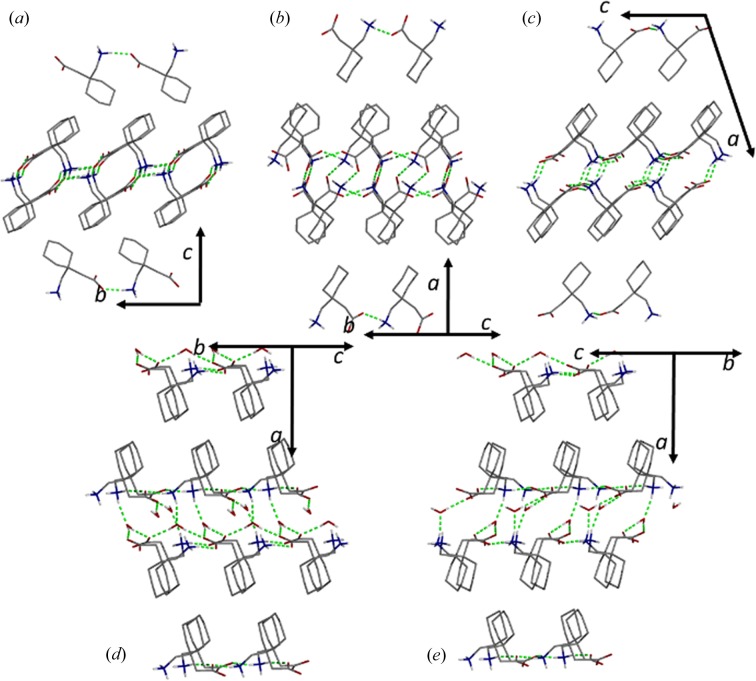
Schematic representation of the crystal packing of the (*a*) GP form α, (*b*) GP form β, (*c*) GP form γ, (*d*) GPH form I and (*e*) GPH form II. Hydrogen-bonding interactions are in green, selected hydrogen atoms have been omitted for clarity.

Like SPG, the structure of GP form α (CCDC refcode QIMKIG) is dominated by 2D networks of charge-assisted hydrogen bonds. In the latter, the bulkier cyclo­pentane group prevents interdigitation of successive layers (Fig. 4 ▸). GPβ (CCDC refcode QIMKIG02) forms 1D chains (Reece & Levendis, 2008 ▸; Wang *et al.*, 2017 ▸; Ibers, 2001 ▸; Vasudev *et al.*, 2009 ▸), whereas the packing in the anhydrous γ form (CCDC refcode QIMKIG03) resembles those of the hydrate forms, GPH I and II (CCDC refcodes QIMKOM and QIMKOM02), and SPGH I and II, the zwitterionic groups being separated into two planes. Remarkably, despite structural similarities, only the hydrates undergo plastic bending, whereas all the anhydrous forms are brittle. This observation suggests that the intercalated water molecules, rather than the weak dispersion forces, are responsible for the observed plastic bending.

## 4. Conclusions   

High plasticity is a desirable property that guarantees processability in crystalline drug materials. To date, different sets of factors have been identified as responsible for plastic crystals, but a universal supramolecular strategy to improve such a property is not recognized yet. For example, it was suggested that bending requires the anisotropic hierarchical distribution of strong and weak supramolecular interactions along perpendicular directions. On the other hand, interdigitation and cross-linked hydrogen bonds (mechanical and chemical interlocks, respectively) have been seen as detrimental for macroscopic plastic bending. At the same time, an increasing number of studies report that, at the microscopic level, hydrated forms exhibit higher plasticity than the anhydrous equivalents. In such phases, increased plasticity does not seem to correlate to a particular structural feature. This is also true for the two zwitterionic drugs SPG and GP. Here, plasticity occurs also at a macroscopic level, on large single crystals, allowing a rationalization for such a phenomenon.

Limited to the investigated systems, the alternation of 2D hydrogen-bonded networks and weak dispersive forces does not guarantee plastic deformation in the anhydrous crystals. In contrast, plasticity occurs in the hydrate forms even when mechanical and chemical interlocks are present. Therefore, in these instances the presence of water appears crucial. Many studies highlight the structural diversity possible for water molecules (Mascal *et al.*, 2006 ▸; Infantes & Motherwell, 2002 ▸; Bajpai *et al.*, 2016 ▸). We speculate that such promiscuity, together with the high mobility of these molecules, enables the quick rearrangement of the supramolecular interactions that are broken by mechanical stress.

Finally we note that SPG and GP are two examples of a wide class of amino acids with biological activity. As sustained by strong and directional charge-assisted hydrogen bonds, the molecular packing of SPG and GP is common to other zwitterionic β-amino­propionic and γ-amino­butyric acids. Therefore, it would be of little surprise if those systems showed the same properties, and the reported observations could be valid for a wide class of drug substances.

## Supplementary Material

Crystal structure: contains datablock(s) I, SPGHI. DOI: 10.1107/S2052252519004755/lq5021sup1.cif

Structure factors: contains datablock(s) I. DOI: 10.1107/S2052252519004755/lq5021sup2.hkl

Structure factors: contains datablock(s) . DOI: 10.1107/S2052252519004755/lq5021sup3.hkl

Supporting figures. DOI: 10.1107/S2052252519004755/lq5021sup4.pdf

CCDC references: 1879470, 1879471

## Figures and Tables

**Table 1:** Crystallographic data for SPGH I and SPGH II

Crystal form	SPGH I	SPGH II
Formula	C_8_H_17_NO_2_, H_2_O	C_8_H_17_NO_2_, H_2_O
CCDC code	1879470	1879471
Temperature (K)	298	150
Space group	*C*2	*P*2_1_
*a* (Å)	9.566 (7)	9.6559 (2)
*b* (Å)	7.440 (6)	29.8416 (6)
*c* (Å)	15.911 (12)	30.1248 (7)
β (°)	101.54 (3)	98.540 (1)
*V* (Å^3^)	1109.5 (15)	8584.1 (3)
*Z*, *Z*′	4, 1	32, 16
ρ (g cm^−3^)	1.061	1.097
μ (mm^−1^)	0.080	0.677
*R*[*F* ^2^ > 2σ(*F* ^2^)] (No. of reflections)	0.0586 (1721)	0.0482 (28820)
