# Supplementary material for: Plasticity in zwitterionic drugs: the bending properties of Pregabalin and Gabapentin and their hydrates
Source: IUCrJ. 2019 May 22;6(Pt 4):630–4. doi: 10.1107/S2052252519004755 (PMC6608624; doi:10.1107/S2052252519004755)
Supplement: Supplementary file 4 [file m-06-00630-sup4.pdf]

IUCrJ

Volume 6 (2019)

Supporting information for article:

Plasticity in zwitterionic drugs: the bending properties of Pregabalin and Gabapentin and their hydrates

U. B. Rao Khandavilli, Matteo Lusi and Patrick J. Frawley

Pregabalin (SPG) and Gabapentin (GP) were purchased from Flourochem. Methanol was purchased from the Sigma-Aldrich and Milli-Q water was used for the experiments.

Crystallization of SPG: SPG crystals were obtained from the commercially available SPG that was dissolved in methanol to saturation and left it at room temperature for 2 days.

Crystallization of SPGH1: SPG was dissolved in pure water and crystallized at 280 K for 3-5 days to get the good quality crystals.

Crystallization of GP: GP and its hydrate crystals are reproduced by following the same procedure mentioned in the reported publications.[1,2]

S1. Experimental Details:

Optical microscopy: SPGH I and GP hydrate crystals were bent with tweezers and a needle, the images have captured using Olympus IX53 microscope under a 4× magnification.

IR Spectroscopy: IR spectra for SPG and SPGH I have been collected on a PerkinElmer Spectrum 100 FT-IR Spectrometer equipped with a PerkinElmer Universal ATR Sampling Accessory.

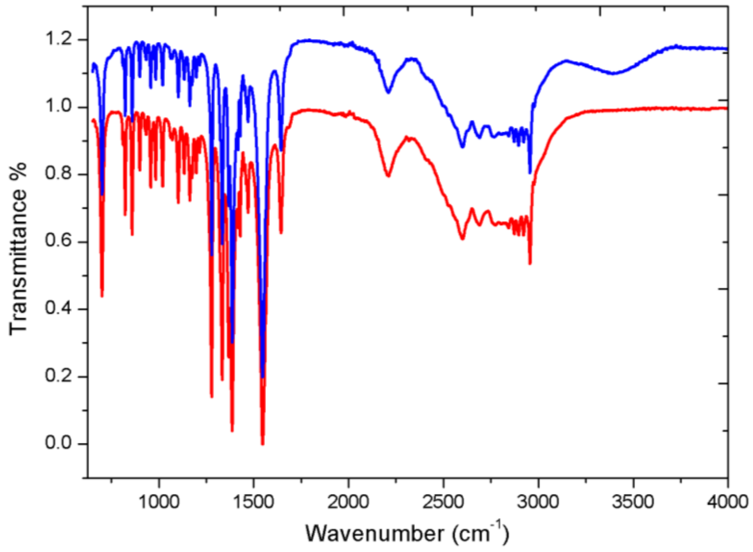

Figure S1 IR spectra for SPG (blue) and SPGH I (red).

Raman Spectroscopy: Raman spectra for SPG and SPGH I have been collected on a Horiba Jobin Yvon LabRam Aramis spectrometer with a 532 nm laser source. The spectrometer was coupled with an Olympus BX40 confocal microscope with a CCD camera cooled by a thermoelectric Peltier device. Raman maps were processed using the LabSPEC 5 software package.

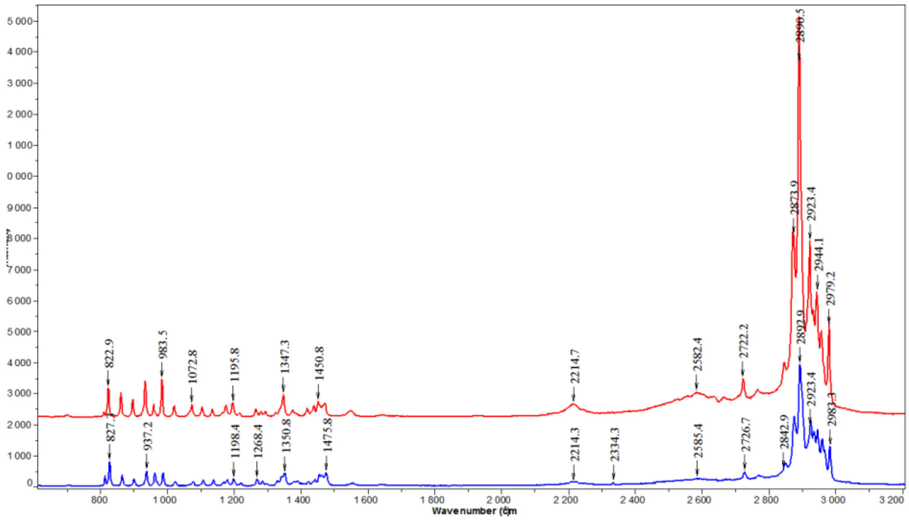

Figure S2 Raman spectra for SPG (blue) and SPGH I (red).

Powder X-ray diffraction (PXRD): PXRD data were collected on Empyrean diffractometer (PANalytical, Philips) using Cu K$\alpha_{1,2}$ radiation (λ = 0.1541 nm) at room temperature operated at 40 kV and 40 mA. The samples were scanned over a range of 4-40° 2θ using a step size of 0.02° 2θ and a scan speed of 0.02° 2θ/s.

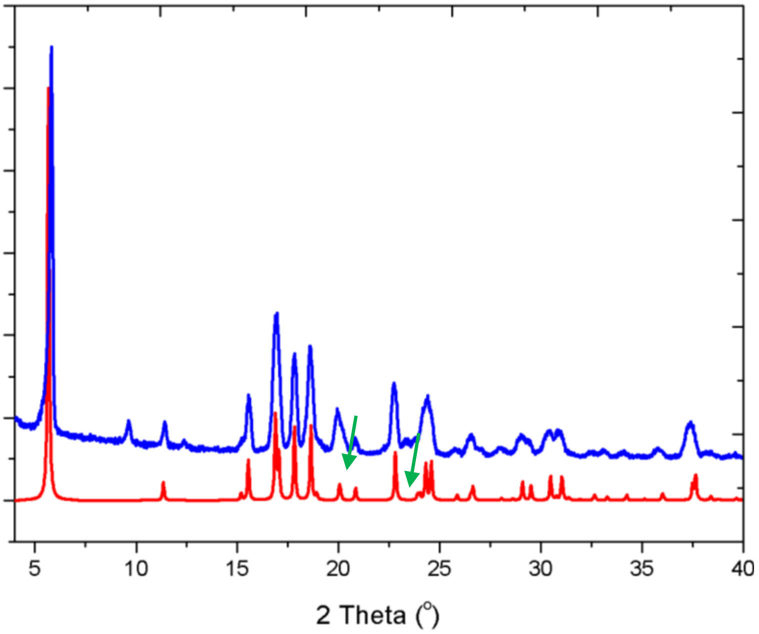

Figure S3 PXRD data for SPGH I: experimental (blue) and theoretical (red), green arrow indicates the traces of SPG.

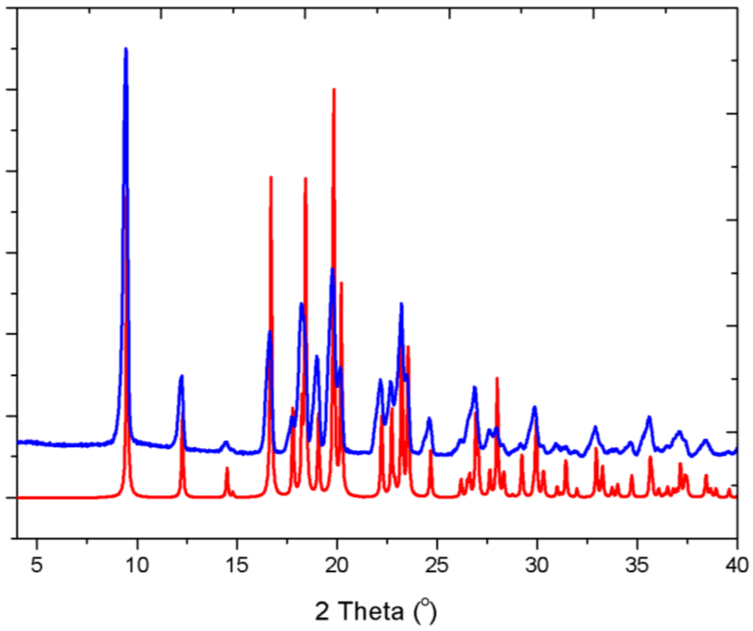

Figure S4 PXRD data for SPG (commercial): experimental (blue) and theoretical (red).

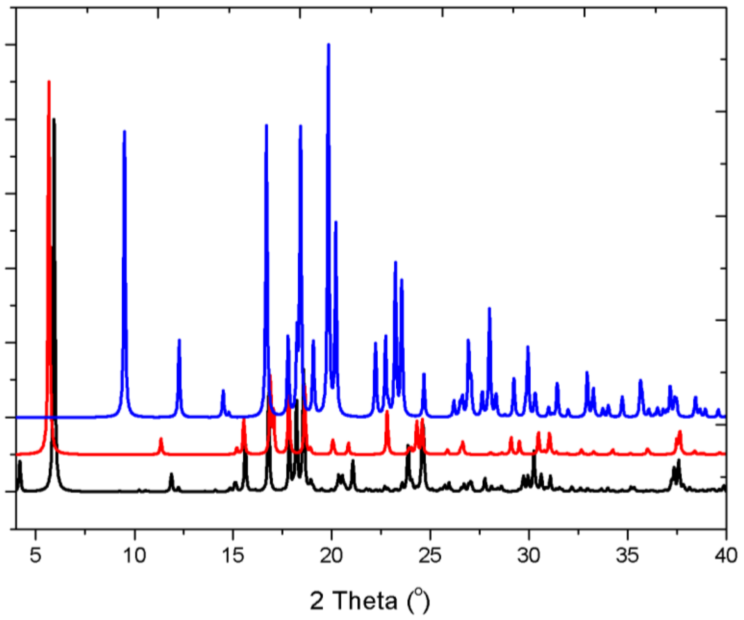

Figure S5 Theoretical PXRD comparison for SPG (blue), SPGH I (red) and SPGH II (black).

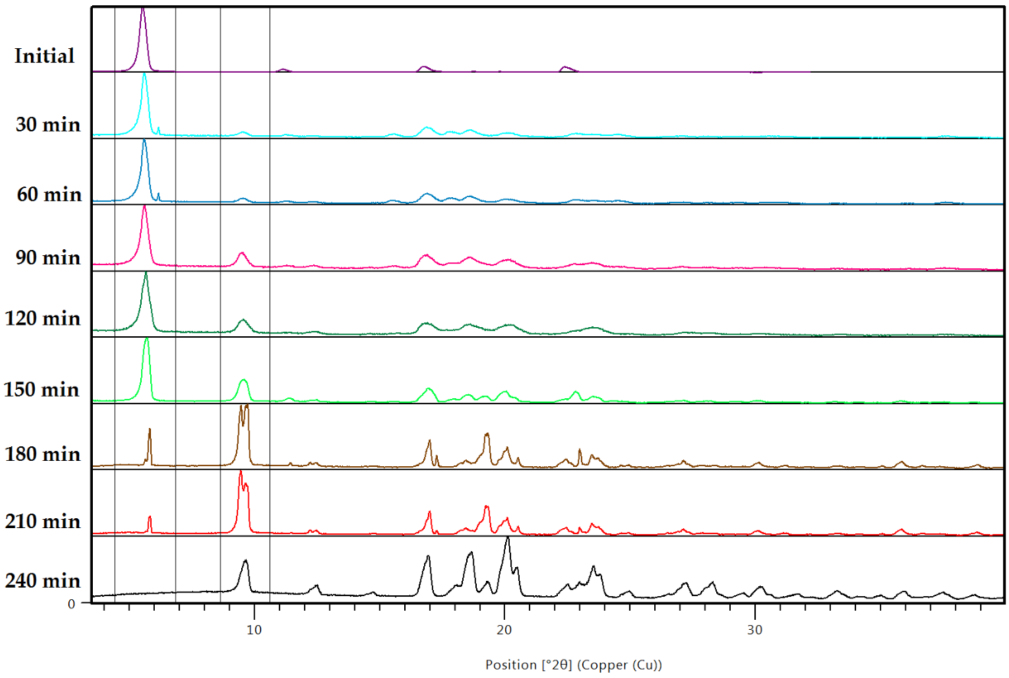

Figure S6 Conversion of SPGH I to SPG with time at room temperature

Differential Scanning Calorimetry: calorimetric measurements of SPG and SPGH I were performed on a DSC 214 Polyma, NETZSCH instrument. Typically, 3-5 mg of sample was accurately weighed into a hermetically sealed aluminium pan and heated to 250 ˚C with 10 ˚C min^{-1} heating rate, under nitrogen gas flow 50 mL min^{-1}.

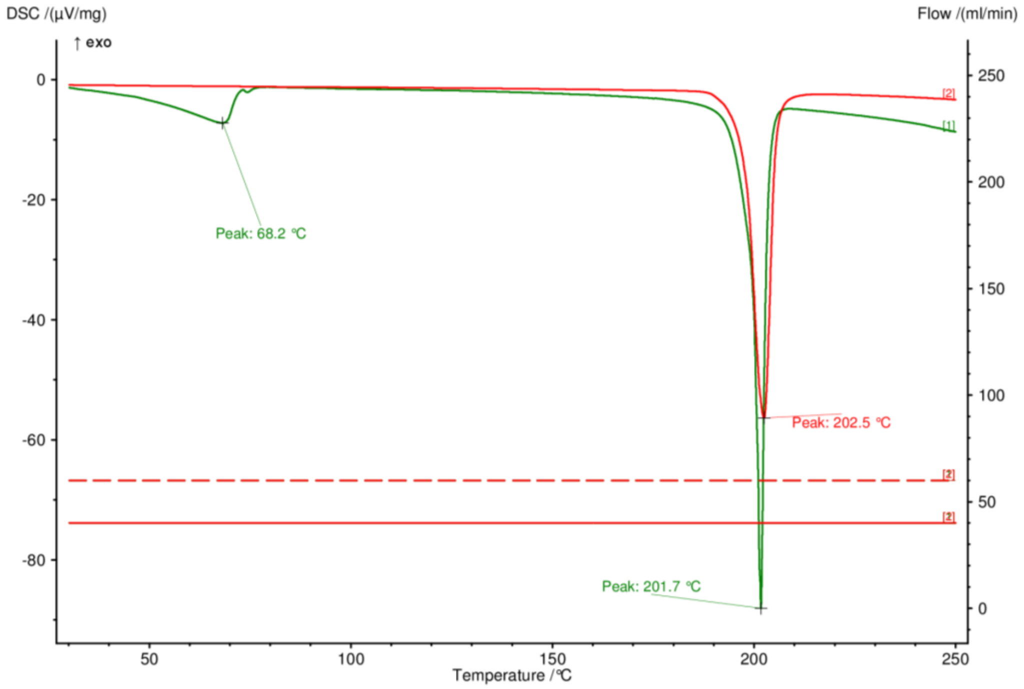

Figure S7 DSC data for SPG (red) and SPGH I (Green)

Thermogravimetric Analysis: Thermograms of SPG and SPGH I were measured with a Perkin-Elmer TGA 4000 with a heating rate of 10 °C min^{-1} under a nitrogen stream of 50 mL min^{-1}.

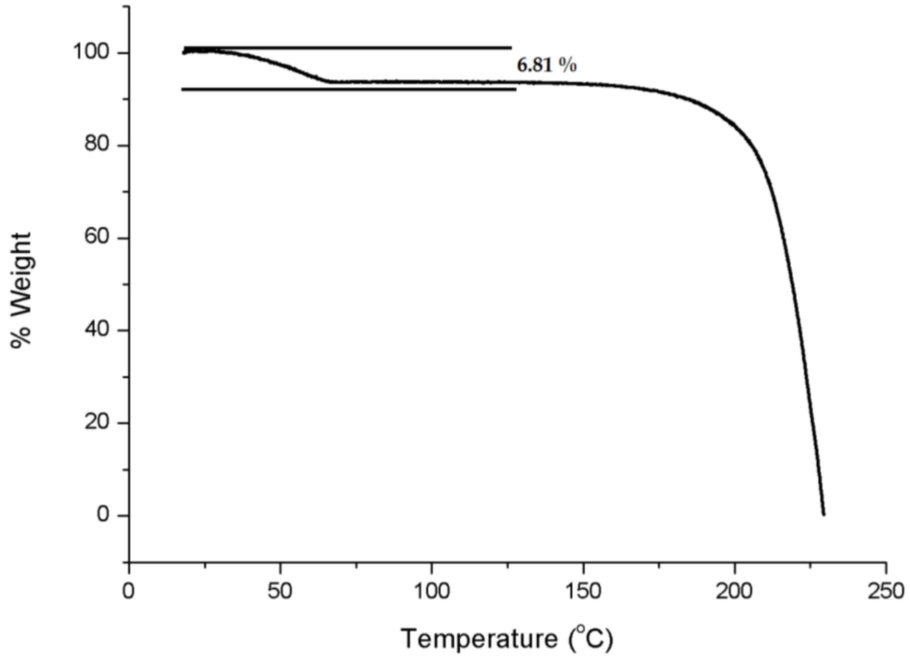

Figure S8 TGA data for SPGH I

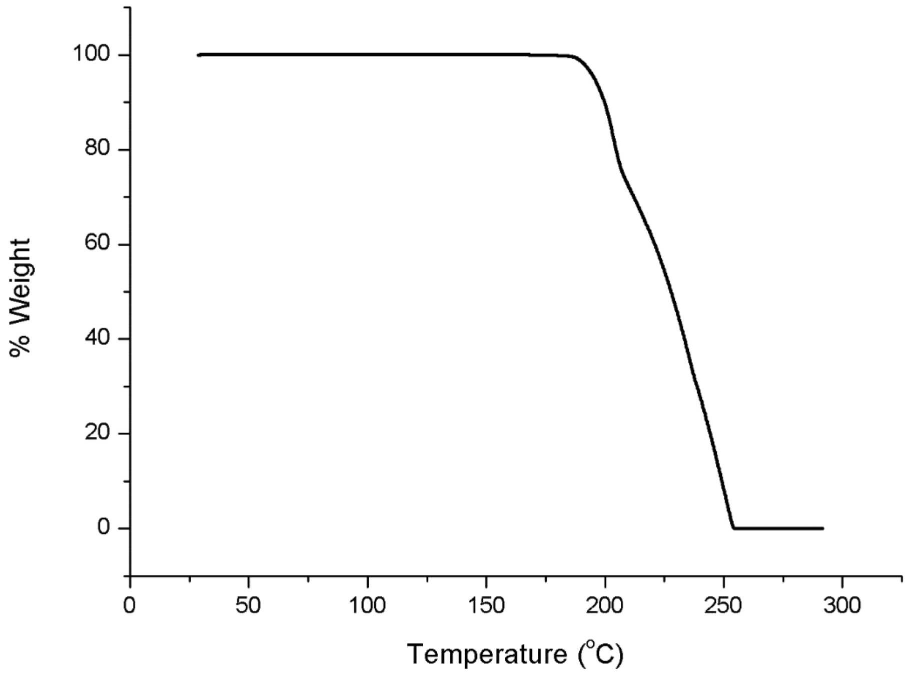

Figure S9 TGA data for SPG

X-ray Crystallography: Single crystal data for SPGH I were collected at ambient temperature using a three circles Bruker D8 Quest with sealed tube Mo anode, Kα radiation = 0.71073 Å, and a Photon 100 detector. Single crystal data for SPGH I were collected at 150 K under a nitrogen-flow (Oxford Cryosystem) using a three circles Bruker D8 Quest with microfocus Cu anode, Kα radiation = 1.5418 Å, and a Photon 100 detector.

The data were integrated and corrected for absorption with the Bruker Apex Suite of programs. The structure solution was obtained by direct methods and refined against all F^2 with the SHELX software interfaced though X-Seed. Non-hydrogen atoms were refined anisotropically and hydrogen atoms were placed in calculated positions refined using idealized geometries (riding model) and assigned fixed isotropic displacement parameters.

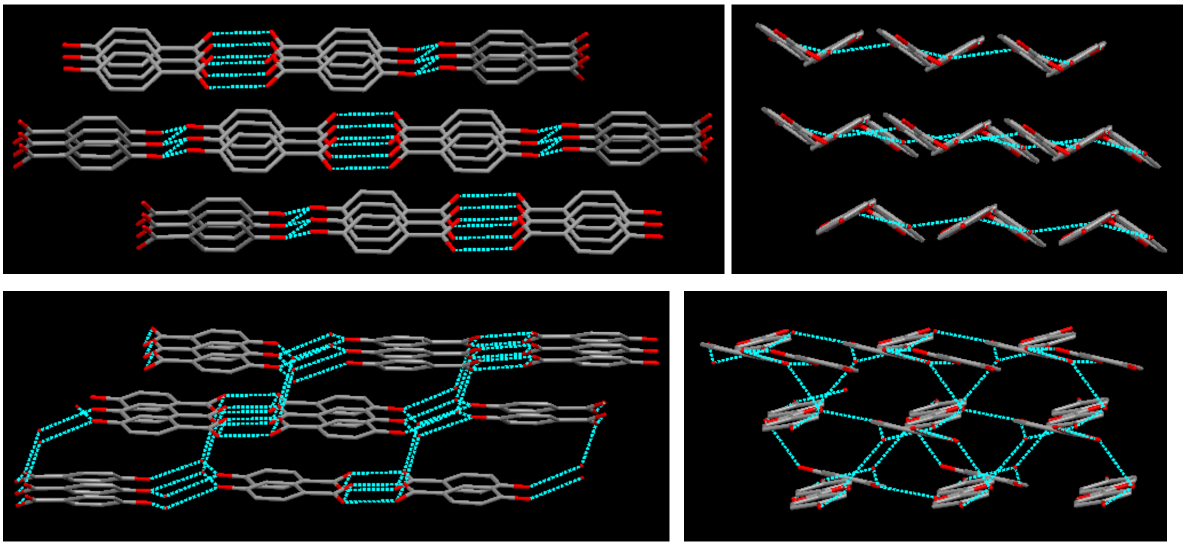

Figure S10 Crystallographic interactions in the *p*-Hydroxybenzoinc acid: anhydrous (top) and hydrate (bottom).

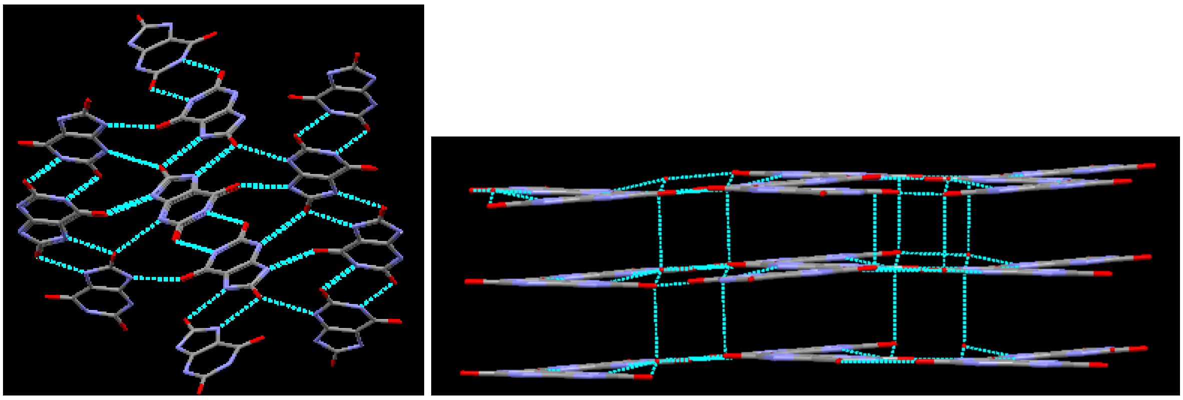

Figure S11 Crystallographic interactions in the Uric acid: anhydrous (left) and hydrate (right).

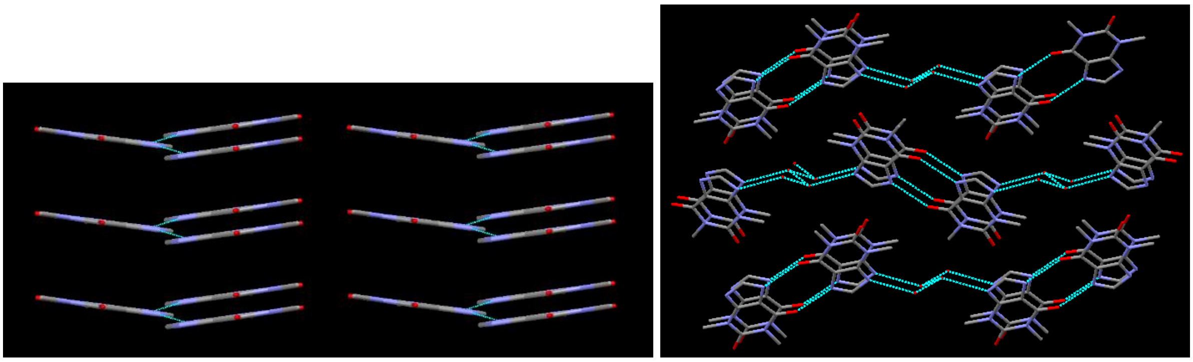

Figure S12 Crystal packing and interactions in Theophylline: anhydrous (left) and hydrate (right).

S2. References:

1. H. A. Reece and D. C. Levendis, Polymorphs of gabapentin. Acta Crystallogr. Sect. C 2008, 64, o105-o108.

2. Y. Wang, S. Du, S. Wu, L. Li, D. Zhang, B. Yu, L. Zhou, H. K. Bekele and J. Gong, Thermodynamic and molecular investigation into the solubility, stability and self-assembly of gabapentin anhydrate and hydrate. The Journal of Chemical Thermodynamics 2017, 113, 132-143.
